# On the Use of Actual Cautery in the Enucleation of Fibroids of the Uterus

**Published:** 1876-02

**Authors:** 


### On the Use of Actual Cautery in the Enucleation of Fibroid Tumours of the Uterus.

Dr. Robert Greenhalgh, in a communication to the Royal Medical and Chirurgical Society (*Medical Times and Gazette*, Nov. 6, 1875), after briefly alluding to the infrequent use of actual cautery in this country, as compared with its application on the Continent, stated that during the last twelve years he had used it frequently, and with more or less success, in chronic enlargements with induration of the cervix uteri due to inflammatory or fibroid disease; in epithelioma and cancer of the neck of the uterus, where the organ is movable; in some cases of vascular tumour of the meatus urinarius; in slight cases of recto- and vesico-vaginal fistula; in incontinence of urine due to dilated urethral canal; and in cases of interstitial and intra-uterine fibroid growths. He expressed an opinion that diffused fibroid deposits of the uterus in the early stage were more amenable to treatment than was generally supposed. He observed that although the large majority of these cases needed no surgical interference, yet there were others which, through the great losses of blood to which they give rise or the mechanical effects they produce, imperatively demand such aid. The author then drew attention to the occasional ill-effects of dilatation and enucleation by the knife, and summed up the advantages of the actual cautery under the following heads: 1, Facility of application; 2, occasions but little pain; 3, rapid in action; 4, occasions no bleeding; 5, no plugging needed; 6, the charred opening not favorable to absorption; 7, no offensive discharge from charred surface; 8, opening readily dilatable and without bleeding; 9, permits of manipulation through opening immediately after its use; 10, by its use portions of the tumour may be rapidly destroyed, its size reduced, and its lower segments rendered conical, thereby facilitating of the opening and the subsequent detachment, expulsion, or removal of morbid growth. He then remarked that spontaneous expulsive efforts shortly followed its use, and the density of the tumours appeared to be more or less reduced after its application. The author, in conclusion, drew especial attention to the three fellowing points: 1. The advisability of the gradual detachment of the growth from its surrounding capsule, especially in in cases where the tumour is of large size, or where the patient has been much reduced by previous hemorrhages, by which further losses of blood are avoided and more perfect contraction of the investing tissues is secured, and the chances of pent-up offensive discharge is almost certainly prevented. 2. The removal of only so much of the tumour at each operation as is external to the opening, by which the opening is kept dilated and all chance of its closure upon the remainder of the growth avoided. 3. The speedy destruction by the cautery or removal by the ecraseur or hand of the tumour should sloughing ensue.—*Medical Abstract.*

